# The comparative recall of Google Scholar versus PubMed in identical searches for biomedical systematic reviews: a review of searches used in systematic reviews

**DOI:** 10.1186/2046-4053-2-115

**Published:** 2013-12-23

**Authors:** Wichor M Bramer, Dean Giustini, Bianca MR Kramer, PF Anderson

**Affiliations:** 1 Erasmus MC - University Medical Center Rotterdam, Medical Library, PO Box 2040, 3000 CA, Rotterdam, The Netherlands; 2 The University of British Columbia, UBC Biomedical Branch Library, Gordon and Leslie Diamond Health Care Centre, 2775 Laurel Street, Floor 2, Vancouver, BC V5Z 1 M9, Canada; 3 Utrecht University Library, PO Box 80125, 3508, TC Utrecht, The Netherlands; 4 University of Michigan, Taubman Health Sciences Library, 1135 E Catherine St, Ann Arbor, MI 48109-5726, USA

**Keywords:** Bibliographic databases, Information retrieval, Systematic reviews, Methodology, Literature searching, Reproducibility

## Abstract

**Background:**

The usefulness of Google Scholar (GS) as a bibliographic database for biomedical systematic review (SR) searching is a subject of current interest and debate in research circles. Recent research has suggested GS might even be used alone in SR searching. This assertion is challenged here by testing whether GS can locate all studies included in 21 previously published SRs. Second, it examines the recall of GS, taking into account the maximum number of items that can be viewed, and tests whether more complete searches created by an information specialist will improve recall compared to the searches used in the 21 published SRs.

**Methods:**

The authors identified 21 biomedical SRs that had used GS and PubMed as information sources and reported their use of identical, *reproducible* search strategies in both databases. These search strategies were rerun in GS and PubMed, and analyzed as to their coverage and recall. Efforts were made to improve searches that underperformed in each database.

**Results:**

GS’ overall coverage was higher than PubMed (98% versus 91%) and overall recall is higher in GS: 80% of the references included in the 21 SRs were returned by the original searches in GS versus 68% in PubMed. Only 72% of the included references could be used as they were listed among the first 1,000 hits (the maximum number shown). Practical precision (the number of included references retrieved in the first 1,000, divided by 1,000) was on average 1.9%, which is only slightly lower than in other published SRs. Improving searches with the lowest recall resulted in an increase in recall from 48% to 66% in GS and, in PubMed, from 60% to 85%.

**Conclusions:**

Although its coverage and precision are acceptable, GS, because of its incomplete recall, should not be used as a single source in SR searching. A specialized, curated medical database such as PubMed provides experienced searchers with tools and functionality that help improve recall, and numerous options in order to optimize precision. Searches for SRs should be performed by experienced searchers creating searches that maximize recall for as many databases as deemed necessary by the search expert.

## Background

For several years, information specialists have discussed which databases (and how many) should be used to perform exhaustive searches of the literature. Prior to 2004, the year of Google Scholar’s release, these discussions focused primarily on traditional databases such as Embase and MEDLINE [1,2]. Further, the general consensus had developed that searching a limited number of databases was insufficient where completeness was the goal [3-6].

In addition, the type of searching that is required to support systematic reviews (SRs) is more complex and time-consuming than searching for simple clinical queries. The demands placed on a searcher for the SR are much higher than for other searches because of the specific requirements of the SR [7] which is integral to science and must therefore be performed systematically, and made repeatable, verifiable and accountable [8,9].

Since 2004, Google Scholar (GS) has been widely-used to locate specific items and aid in cumulating the scholarly literature. In 2005, Giustini [10] stated that GS produced acceptable results for browsing routines but results of low precision meant that its use for other searching was problematic. Since then, GS has improved its scope; from 2005 to 2012, its coverage of the literature rose from 30 to 88% to 98 to 100% [11,12]. An important unanswered question about GS remains: 'Is GS advanced enough in its development to replace more sophisticated tools such as PubMed or Embase?’

In 2007, Shultz [13] provided an overview of criticism of GS that had been generated since its debut in 2004. Unfortunately, many of the original shortcomings identified between GS and *traditional bibliographic* databases such as MEDLINE and Embase are still in evidence: GS lacks a controlled vocabulary, search histories and sets cannot be built and manipulated and wildcards and limits (for instance study types) cannot be used precisely. Only the first 1,000 citations of any search in GS are viewable and search strings must be kept under 256 characters.

Since 2004, a number of studies have examined the value of GS in biomedical searching. Falagas, Pitsouni*, et al.*[14] compared four databases including GS and PubMed, and concluded that GS retrieved more obscure items than other search tools. Anders and Evans [15] focused on using advanced searching in GS and PubMed but, given major differences in the databases, their study made true comparisons difficult. In Nourbakhsh, Nugent*, et al.*[16], researchers found that the first 20 results in GS often produced more relevant hits than similar searches in PubMed. But since PubMed, until very recently, lists citations in chronological order (not by algorithms, as in GS) the authors’ conclusions are counter-intuitive. The frequently cited study by Walters [17] covered one topic (on older person migration), which is out-of-scope in a medical database. A recent study by Shariff, Bejaimal*, et al.*[18] compared search strategies designed by end users and compared the first 40 hits in GS and PubMed.

In 2013, Gehanno, Rollin*, et al.*[19] published a paper that generated important critical discussion of the value of GS. In their paper, Gehanno *et al*. used GS to locate all studies originally cited in a published SR. By finding all known items, the authors argued that GS, after some improvements to increase its search precision, could be used alone in searching for SRs.

The article by Gehanno *et al*. drew much attention to GS and resulted in some follow-up articles. Giustini and Kamel Boulos [20] argued that a 'known-item’ searching is a very different activity than locating articles by subject, as is attaining 100% recall of the (mostly unknown) relevant literature. Boeker, Vach*, et al.*[21] reinvestigated the results of Gehanno *et al*. using search strategies designed to match Medline strategies used for Cochrane systematic reviews. However, the authors designed the searches themselves and failed to account for the maximum number of results that can be retrieved in GS (1,000), although they mentioned this limitation in their manuscript. The low precision in GS as reported by Gehanno *et al*. and by Boeker *et al*., is mainly an artifact due to the large number of hits that are reported by GS. Since search results cannot be viewed beyond the first 1,000 references, actual precision in GS should be calculated as the number of relevant references found in those first 1,000 references, divided by the number of hits that can actually be viewed, which is 1,000 at most.

The research done on GS for SRs is limited in methodology, which is crucial for evaluating GS for SR searching. In Table 1 we critique in more detail earlier research on GS.

**Table 1:** Limitations of current published research on the usability of Google Scholar for medical purposes

**Limitations of research**	**References**
Not testing for systematic reviews	[13-16,18,22-26]
Limited number of searches	[14-16,24-26]
Relevancy of results only determined by the authors	[13,14,16,23,25,26]
Not reviewing the first 1,000 hits in Google Scholar	[15,16,18,19,21,22]
Only using searches designed by the authors	[14-16,21,22]
Searches not comparable between the databases	[15,16,23,25]
Published more than five years ago	[13,14,23,24]
Only searching for known items	[13,14,19]
Only looking at coverage, not retrieval	[19]

Though it seems unlikely that an experienced information specialist would use GS as the sole database in a SR, a less experienced researcher, faced with the enormous task of performing a review without expert help, might be tempted to do so (based on the aforementioned research). At least one review is known that, after doing preliminary searches in a wide range of databases decided ultimately to use only PubMed and GS, but failed to notice their search strategy was not executable in GS, since it was over 500 characters long [27].

In this paper, the usability of GS in searching for SRs is considered, where relevancy is pre-determined by inclusion in papers that have been previously published. Both the original (identical) topical searches reported by those papers and searches improved by an information specialist are used in order to compare the recall within GS and PubMed. The aim of this paper is to discover whether the original authors would have found all included references by using GS only. When studies from the original SRs were not found, it is assessed whether a more exhaustive search strategy created by an information specialist would improve recall. Given its potential for one-stop searching, we assess whether GS can indeed replace the multiple databases required for the SR and locate all studies needed to conduct a SR.

## Methods

In May 2013, PubMed and Embase were searched using the exact phrases 'systematic review’ and 'google scholar’ in title and/or abstract fields. Of the records identified, the full-text of relevant papers was retrieved on the open web or via subscriptions at the first author’s institution. The full-text and appendices of available articles were scanned for descriptions of the strategies used to search PubMed and GS.

Articles that clearly described identical search strategies were investigated further. The queries as performed in the initial searches in the SRs were recreated. If the SRs did not discuss a medical topic, the review was excluded as it was unlikely that PubMed would have been viewed as a valuable database in those instances. When the length of a reproduced search exceeded the maximum query length allowable in GS (256 characters) the review was also excluded. All inclusion and exclusion criteria are summarized in Table 2.

**Table 2:** Inclusion and exclusion criteria

**Inclusion criteria**	**Exclusion criteria**
Systematic review in a medical topic	Length of search strategy greater than 256 characters
Reporting the use of both Google Scholar and PubMed	Number of hits retrieved in PubMed exceeds the reported total number of hits reviewed
Reporting in reproducible detail an identical single phrase search for these databases	

Searches that were reproducible were executed in both GS and PubMed, and the number of hits was documented accordingly. In PubMed, results were limited to before the MeSH date (field: [mhda]) of the original search date as stated in the article. The number of hits in PubMed was compared with the number originally reported (either for PubMed, or the total for all databases). If it did not exceed that number, the SR was included. In GS, search results were limited to the publication start year used by the original authors and the end publication year of the search date. Because publication dates can differ from search dates (because publication dates are generally added to the print version, while the electronic version might be available longer) we checked whether the list of includes contained articles with newer publication dates, and if so, changed the publication limits accordingly.

For each replicated search, the first 1,000 results of GS were saved in a Word document. Using the 'find’ function in Word, occurrences of each included reference from the original SR were identified. Distinctive fragments of the title were searched but where no match was located, author names were searched. If a citation was not found among the first 1,000 results, GS coverage for that item was checked using author names and part of the title between double quotes. If the item was indeed present in GS, the reference was checked for retrieval in the search query (beyond the first 1,000 hits) by combining author names and distinctive title words with the full query (to check whether they had ranked low on Google’s PageRank algorithm).

We did not exclude hits that were citations only (by definition) but for those references, it was checked whether the citing articles, as linked in GS, were published before the citing systematic review. It could then be assumed that the citation was present in GS when the original authors performed their searches. If the citation was only present in articles with a more recent publication year, the result was confidently discarded.

All included studies were searched in PubMed by searching for the complete reference. If PubMed did not reveal a match, a second attempt was performed using a combination of first author [1au], page number [pg] and publication year [dp]. Included references were collected using the PubMed Clipboard. Once all references were retrieved, clipboard contents were checked against the results of the replicated search.

The intention of this project was not to judge the quality of the replicated searches. In a later stage, we improved some searches to investigate whether more citations could be found. An experienced information specialist (WB) created improved search strategies for GS based on the original authors’ description of their research question, without taking into account the included references from that SR. A second search strategy was designed based on the frequency of words in the titles of included references of these SRs. For the searches that had missed the most included references in PubMed, an information specialist created a more comprehensive search strategy using MeSH terms and free text, without using the included references to determine search words.

## Results

**Figure 1:**
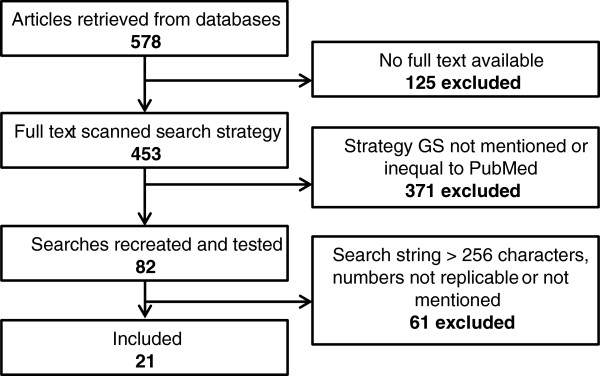
**Flow diagram of reviewed articles.** Bramer *et al*. - the recall of Google Scholar is insufficient.

Of the 578 SRs retrieved, the full-text was obtained for 453 articles. A total of 84 articles described in enough detail identical searches that could be rerun in PubMed and GS. Eight articles were excluded because their search strategies exceeded the maximum search length allowed by GS (256 characters). Twenty articles were excluded because they made no mention of the number of hits retrieved. Two articles were excluded because the topic was non-medical and therefore their search strategies returned no results in PubMed. Seven articles were excluded because the authors used multiple search queries and 24 others were excluded because the numbers reported (for PubMed or total) did not match number of hits retrieved for the replicated searches. See Figure 1 for a flow diagram of the in- and exclusion procedure. For one article the list of included references contained three references from beyond the search year, thus we decided to expand the publication date limits with one year.

In 21 articles, the cited searches for both GS and PubMed were identical, well-documented, and the number of hits in PubMed did not exceed the number of hits first reported. Additional file 1 describes the original and replicated searches along with other parameters and the detailed results. In eleven cases, the searches used in this research were exactly the same as those described in the full text or appendices of the studies. In ten instances, some minor changes had to be made. In some cases, Boolean operators (AND and OR) were not stated and nesting was not clearly laid out; proper searches cannot be performed without operators but what was intended was clear. If major changes were required, the reviews had been excluded. For five references retrieved as citations, the citing articles all had a publication date later than the original search date, so these citations were ignored. A full list of references to all SRs included in this article can be found in Additional file 2.

### Coverage

The total number of studies included by the SRs was 541. In GS, ten studies were not present, thus the overall coverage of GS reached 98%. In PubMed, 48 references were not present, so PubMed had an overall coverage of 91%.

### Recall

Of the total number of included studies that were reviewed (541), 389 (72%) were present in the first 1,000 hits of the original searches in GS. Forty-five articles had been retrieved by the search strategy in GS, but were not among the first 1,000 hits. If GS had allowed its users to review all search results, recall would thus have been 80%. The same searches retrieved 369 hits in PubMed (68%).

### Precision

**Figure 2:**
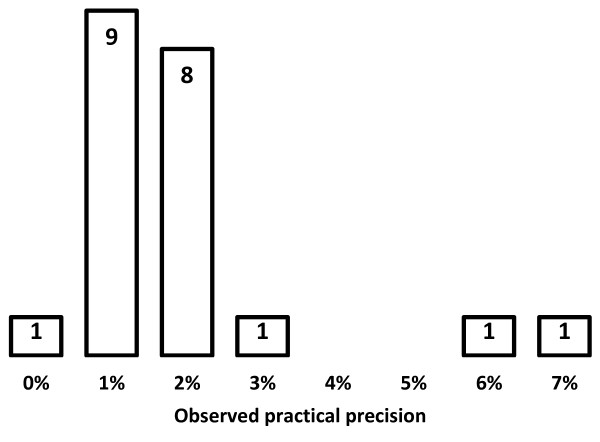
**Practical precision of Google Scholar.** Bramer *et al*. - the recall of Google Scholar is insufficient.

As shown in Figure 2, practical precision in GS has an average of 1.9% and a median of 1.7%. Average precision for SRs, according to Sampson, Tetzlaff*, et al.*[28] is around 2.9%. The practical precision in GS for the searches observed in this article is slightly below the reported average but 1.9% is nonetheless quite acceptable for SR searching, where, in order to be complete, researchers must browse through irrelevant hits to find important references.

### Improvement of search strategies in Google Scholar

163 included studies were not present in the first 1,000 hits of the original searches in GS. For five SRs where GS had missed more than ten included references (in total 110), we tried to improve the search strategies. Using the first improved search strategies, created without taking into account the included studies, retrieval in GS for these five SRs increased from 53% to 60%. The search strategies designed to capture as much included studies as possible (when the search strategies were based on words in the title of the included studies) resulted in 66% retrieval for these five SRs (Table 3). The improved search strategies for GS are available in Additional file 3.

**Table 3:** Systematic reviews of which more than ten included references were not retrieved in Google Scholar; performance of improved searches

	**Number of included references**	**Number of includes retrieved by**		
		**Authors’ search**	**Improved search #1**	**Improved search #2**
Hasani	78	26	26	37
Novak	30	10	12	12
Verhoeven	89	72	75	72
Navarese	17	6	13	16
Belsey	20	10	15	16
	234	124 (53%)	141 (60%)	154 (66%)

### Improvement of search strategies in PubMed

Of the references found in PubMed, 124 were not retrieved by replicating the original searches. Of these, 111 were included by the seven SRs in Table 4. The other SRs each had less than three included references that were not found in PubMed. We tried to create better searches for the research questions of these seven SRs to see if this would increase retrieval in PubMed. Using the improved search strategies increased retrieval for the seven SRs from 61% to 85%. The improved strategies for PubMed are available in Additional file 4.

**Table 4:** Systematic reviews that contributed most to the 'not retrieved articles’ in PubMed; performance of improved searches

	**Number of included references**	**Number of includes retrieved by**	
		**Authors’ search**	**Improved search**
Javan	68	29	61
Hasani	78	51	67
Verhoeven	89	67	81
Navarese	17	5	5
Novak	30	19	26
Gupta	16	8	12
Hardefeldt	26	18	23
	324	197 (61%)	275 (85%)

## Discussion

Literature searching in multiple databases can often be cumbersome and is always time-consuming if it is done well. GS offers an easy-to-use, familiar interface and relevance ranking, making simple searching for a few good articles much easier. However, the use of GS as a robust search tool is not without its challenges.

To focus on the differences between the databases, the research was restricted to SRs that used both GS and PubMed with identical search strategies. Although this research represents a small sample of all published SRs, and is not representative, we nonetheless believe our findings to be of indicative of a trend. Had SRs been selected that did not describe their GS search strategies, it would have been necessary to create them which was not the purpose of this research. If SRs reported non-identical searches for GS and PubMed, the findings would be reviewing the ability of the original reviewers to translate their searches, which again was not the intention.

One of the most challenging aspects is the frequency with which Google changes its functionality without giving any prior notice to its users. In 2012, GS changed its advanced searching features and removed the ability to limit results to specific domains and disciplines (for instance medicine). In March 2013, GS reduced the maximum number of articles it can show per page from 100 to 20, and in June the tilde operator, that was very usable to search for synonyms (frequently used as a replacement for truncation), was removed from the regular search engine (google.com), at the moment it is still available in GS. These changes have a major impact on searching, and when users asked whether some of these features would be reinstated, Google said little [29]. Even more threatening is the fact that since the end of 2011, GS has disappeared from the menu of regular Google, thus making it harder to find for those users who do not already know of its existence, although results from GS and a link to a search in GS ('Scholarly articles for…’) often appear in the search results of regular Google. Because of Google's tendency to shut down applications it considers less frequently used (like recently Google Reader in June 2013), this might be a threat for the continuity of GS. And if GS were to be shut down, this would be a major threat to the replicability of the methodology of the SRs that were performed with a GS search.

Many published SRs report on the total number of hits in the databases they have used. However, the ratio between the number of hits we retrieved in PubMed and the total number of hits that were reported by the original authors varied a lot. In one case the reported total was 108 times higher than the number we found in PubMed, while for many other SRs the ratio between numbers reported and what was retrieved was equal to one. Some authors opted to report the total number of citations found in GS, while others took into account the first 1,000, or just reported the number they felt was necessary to view. Still, others ignored number of hits in GS in their reporting and counted only relevant, unique hits. Consequently, the resulting numbers in our searches were at odds with what the original authors reported in their published reviews. It is recommended that authors of SRs that use GS as a primary source report only those hits that were actually reviewed from GS.

Search reproducibility was low due to inaccurate or incomplete reporting of search strategies. Many papers that were examined referred to their search strategies by listing the keywords and Boolean operators used in an illogical order [30]. Even in cases where searches were explicitly stated, the number of hits did not match the number of hits retrieved using the exact limiters and search parameters. To ensure transparency and reproducibility, authors of SRs should take care to follow the guidelines in the PRISMA Statement [7] for reporting search strategies. This states that the number of studies screened should be stated and not the number a database claims to have found.

Importing all references into reference management software is now a standard feature in bibliographic databases such as PubMed and Embase. GS does not offer such a feature. With Zotero, all results from one page can be imported from GS. However, recently the maximum number of hits shown per page changed from 100 to 20, making downloading the full set of hits more time consuming. When the authors of this article used Zotero to import the contents of a single search into Endnote, after downloading 200 references a 'Captcha’ was shown, as Google had detected that our 'computer or network may be sending automated queries’. GS seemed disinclined to provide the flexibility required to properly search the literature for the SRs.

We observed that many items were found because GS indexes content beyond the abstract into the full-text of articles, including their references. Excerpts often show a part of the article containing the reference list with the searched words found in titles of referred articles. When searching for included references in the first 1,000, numerous false hits were encountered showing the included articles in the reference list of other retrieved articles. GS seems to perform citation tracking for articles by using search words in the title. This accounts for much of the extra hits (or 'noise’) in GS’ results.

One limitation in this research (as in all retrospective research involving GS) is one can never be certain of GS’ coverage at any specific point in time, which is a serious problem for searchers. We performed searches several years after the original reviews were performed (average search date was September 2010 but ranged from Jan 2007 to October 2012, while we searched GS in May/October 2013). Although we limited GS searches to the publication year of the original search date, the results will probably differ from those retrieved by the original authors. Not surprisingly, the number of hits retrieved during our searching was often at odds with what was originally reported. Since replicable searching is essential in performing research, the inability to reproduce searches in GS severely limits its value to researchers. Bibliographic databases such as PubMed offer additional database management dates next to publication dates. These databases keep track of content, and note when changes are made. In PubMed using the field mesh date ([mhda]), one can be rather certain what a search result would have been on a given date. GS lacks these management dates, and only offers publication dates. Because of the absence of a clear date restriction feature, replicability, the search process (which is crucial to SR searching) is very is rendered far more problematic than would be the case in curated databases such as Medline and Embase.

We limited our searches to the publication dates of the last search date. However, this reduced the reported hits substantially. Even when a theoretical limit to publication years 1800 to 2099 was applied, the number of hits dropped. In simple queries, this seemed to affect only the number of hits, but not the resulting references. The first 1,000 articles remain largely the same, making this a good replacement for date limits in bibliographic databases. However, on the improved search strategies that were more complex, the number of hits reported often dropped a factor ten, even with the theoretical limit to all publications dates. Resulting references differed immensely, and the first 1,000 hits hardly contained any of the included references. Therefore, for the improved searches, publication date limits were not used. This is a newly identified problem when using GS for SR searching.

Though it was not our intention to judge the quality of the searches of the SRs, we believe that the quality of the searches was poor as they often only combined a few words with hardly any synonyms. This is of course also due to the selection process: if an identical search is used in GS, none of the more sophisticated tools of PubMed (for instance MeSH terms, field codes and truncation) could have been used.

Improving search strategies is a major challenge in GS. As GS cannot store search histories, it is mostly impossible to build multi-set queries or evaluate changes made to search queries. Search strings are momentarily limited to 256 characters, and searchers have to select their keywords accordingly. This is further complicated by the fact that GS does not allow truncation as required by searchers. A feature that could replace truncation in GS is the tilde (~), which automatically searches for word variants. Although useful, the feature does not work in combination with Boolean operators like OR, and therefore cannot be used in exhaustive search queries. In addition, the feature was recently deprecated from Google's regular search engine, making its continued availability in GS uncertain. Using | instead of OR and by simply leaving out ANDs it is possible to use as many of those characters for search words as possible. Finally, GS has no feature for proximity or adjacency searching; parentheses can be used to search word variants in a double-quoted phrase ("(myocardial|heart) (infarct|attack)"), as well as asterisks (*), but the number of asterisks used marks the exact number of words allowed, where proximity searching in other databases generally describes a range of words. These missing features make a translation of a proper search query as designed for other databases difficult if not impossible. The limitations experienced in this research are presented in Table 5.

**Table 5:** Comparison of Google Scholar and PubMed in systematic review searching

**Google Scholar**	**PubMed**
Shows up to 1,000 results	Shows all results
Searches the full text of the article, and words on the webpage	Searches only bibliographic data and controlled vocabulary (MeSH terms)
No controlled vocabulary available	Controlled vocabulary (MeSH terms) added by skilled indexers and searchable (including 'explode’)
Searches the broad aspect of science (filters limiting results to medical articles were removed)	Only contains articles on medical topics
No search history available (unable to compare or combine record sets)	Detailed search history available, flexibility in combining record sets to create complicated search strategies
Search queries limited to 256 characters	No limits on the length of search queries
No truncation allowed. Tilde can be used to search for variants, but cannot be used in OR relationship with other words. GS is said to search for word variants, but this is very rare and the mechanism is unclear.	Truncation allowed
Possibly automatic searching for synonyms (details unclear)	Automatic Term Mapping (details available)
Field names only for title (complete query) and author names	Field names for many fields can be assigned per synonym
No advanced limits (for publication type, human studies and so on)	Multiple advanced limits in the database itself, or available from third parties
Cannot accurately limit to search dates (no controlled updates)	Different date fields available to limit searches to results before a certain date
Cannot download results in bulk to reference management software	Multiple options to download the complete results set to reference management software
Proximity searching only with exact order and exact number of connecting words	No proximity search possible

Though we wanted to improve the searches that returned the least included references, we could not draw conclusions about the effects of our improvements. We do not know, for example, if the authors missed important references in their original searches. We assume that within the extra hits retrieved, extra relevant studies might have been found. Because GS was unable to retrieve all articles found by the authors, its results cannot be considered complete.

## Conclusion

As we've shown, the coverage and precision of GS are acceptable. Although coverage is not 100%, for many investigators 98% might suffice for simple literature or narrative reviews of a topic. The overall precision of GS (using the total reported number of hits as denominator) is rather low, but the practical precision, calculated by the number of relevant hits in the first 1,000, with 1,000 as denominator is theoretically acceptable for SR searching, and highly dependent on the number of included references.

Our *a priori* question was 'Is GS’s recall sufficient to be used on its own in systematic review (SR) searching?’ Overall retrieval in GS is 72%, which is too low for it to be used as a single database for the SR. PubMed fared similarly at 68%. The creation of better searches in GS proved to be a constant challenge. A high of 66% recall was achieved for the five searches that initially missed the most references from the SRs. In PubMed, our improved searches reached 85% recall. Neither database was sufficient on its own to find all articles from previously-published SRs.

Researchers from other disciplines might find results in GS that are 'good enough’. Similarly, medical professionals might find using PubMed (or another bibliographic database) on its own is good enough for day-to-day searching. Some may even prefer GS for initial searching to find 'a few good articles’ due to its excellent relevance ranking.

However, SRs require a complete view of all existing literature in a given area. This can only be achieved by performing exhaustive searches of relevant databases and websites in consultation with a trained information specialist. These important searches that support the SR methodology must be repeatable, verifiable and accountable, which poses a problem with GS.

There is therefore no reason why GS should be viewed as more suitable for performing SR searches than PubMed (or any other specialized database). We hope that our research will inform future authors and guide their use of GS. Authors of future SRs should continue to use GS but in concert with multiple other databases, not as a replacement of other databases.

## Abbreviations

GS: Google Scholar; SR: Systematic review.

## Competing interests

The authors declare that they have no competing interests. No funding has been received for this research.

## Authors’ contributions

WB designed the research, reviewed the included SRs, collected the data and optimized the final searches. DG, BK and PA provided feedback on the initial results and interpretation of the data. WB prepared the manuscript with revisions from DG, BK and PA. All authors read and approved the final manuscript.

## Authors’ information

WB is an information specialist at Erasmus MC, Rotterdam University Academic Hospital. He holds a BSc in biology and information science. He performs exhaustive searches for more than 200 systematic reviews a year.

DG is the UBC Biomedical Branch Librarian at Vancouver General Hospital in Canada. He holds MLS and MEd degrees.

BK is an information specialist at Utrecht University Library, working at the University Medical Center Utrecht. She holds a PhD in Neurobiology.

PA is Emerging Technologies Librarian at the University of Michigan. She holds an MLIS and a BSc in psychology and music.

## Supplementary Material

Additional file 1 **Search strategies replicated from included articles and obtained results.** A full table of the original description of the searches performed by included SRs, the searches as we used them, restrictions of the original searches (that is, search date, and start year) and number of included references. Click here for file

Additional file 2 **Systematic reviews included in this article.** A reference list of all systematic reviews used in this article. Click here for file

Additional file 3 **Improvement of search strategies in Google Scholar.** A description of the improved search strategies for Google Scholar and the results obtained with them. Click here for file

Additional file 4 **Improvement of search strategies in PubMed.** A description of the improved search strategies for PubMed and the results obtained with them. Click here for file
