# Supplementary material for: The comparative recall of Google Scholar versus PubMed in identical searches for biomedical systematic reviews: a review of searches used in systematic reviews
Source: Syst Rev. 2013 Dec 23;2:115. doi: 10.1186/2046-4053-2-115 (PMC3882110; doi:10.1186/2046-4053-2-115)
Supplement: Additional file 1 — Search strategies replicated from included articles and obtained results. A full table of the original description of the searches performed by included SRs, the searches as we used them, restrictions of the original searches (that is, search date, and start year) and number of included references. [file 2046-4053-2-115-S1.pdf]

Search strategies replicated from included articles and obtained results

First author	Original search description	Search strategy	Changes	Original search date	starting year	Hits GS	Hits PubMed	Included items	Includes in GS (1000)	Includes in PubMed
Belsey	(constipation) AND (randomised OR randomized) AND (PEG OR polyethylene OR macrogol OR movicol OR idrolax OR miralax OR transipeg OR forlax OR colyte OR golytely OR isocolan OR nulytely) NOT colonoscopy	(constipation) AND (randomised OR randomized) AND (PEG OR polyethylene OR macrogol OR movicol OR idrolax OR miralax OR transipeg OR forlax OR colyte OR golytely OR isocolan OR nulytely) NOT colonoscopy	Exact	1-10-2009	1970	1630	57	20	7	14
Bomback	“membranous” and “rituximab.”	membranous AND rituximab	Exact	1-8-2008		18800	49	21	17	18
Cassie	“preoperative weight loss” AND “bariatric” OR “obesity”	"preoperative weight loss" AND (bariatric OR obesity)	Added necessary parentheses	1-1-2011		931	47	26	21	16
Deeba	obturator hernia AND laparoscopic	obturator hernia AND laparoscopic	Exact	1-12-2009	1991	2900	51	17	17	17
Donnenwerth	“microfracture” AND “ankle” OR “talus”	microfracture AND (ankle OR talus)	Added necessary parentheses	1-1-2012		5600	31	7	7	6
Espinosa	“Hispanic, Hispano, Hispana, Latino, or Latina,” and “fatalism, fatalismo, or fatalistic beliefs” and “cancer screening or cancer.”	(Hispanic OR Hispano OR Hispana OR Latino OR Latina) AND (fatalism OR fatalismo OR fatalistic beliefs) AND (cancer screening OR cancer)	Added necessary parentheses and Boolean operators	25-2-2010		4000	36	11	9	7
Gougoulias	‘calcaneal’ and ‘fractures’,	calcaneal AND fractures	Exact	5-12-2008		24700	2027	6	6	6

First author	Original search description	Search strategy	Changes	Original search date	starting year	Hits GS	Hits PubMed	Included items	Includes in GS (1000)	Includes in PubMed
Gupta	fesoterodine, overactive bladder, and muscarinic antagonists	Fesoterodine AND overactive bladder AND muscarinic antagonists	Added necessary Booleans	1-12-2009		528	21	16	9	8
Hardefeldt	‘‘thyroid disease’’ or ‘‘hyperthyroid’’ or ‘‘hypothyroid’’, ‘‘thyroiditis’’ or ‘‘graves’’ AND ‘‘breast disease’’, or ‘‘breast carcinoma’’, or ‘‘breast cancer’’ or ‘‘breast neoplasm’’	(“thyroid disease” OR “hyperthyroid” OR “hypothyroid” OR “thyroiditis” OR “graves”) AND (“breast disease” OR “breast carcinoma” OR “breast cancer” OR “breast neoplasm”)	Added necessary parentheses	1-12-2011		19200	188	26	22	18
Hasani	“obesity” and (“herbal medicine” or “plant”, “plant medicinal” or “medicine traditional”)	obesity AND ("herbal medicine" OR plant OR "plant medicinal" OR "medicine traditional")	Added necessary Booleans	1-1-2009		109000	699	78	20	51
Jahangiri	PubMed: (IBS[All Fields] OR (“irritable bowel syndrome”[MeSH Terms] OR “irritable bowel syndrome”[All Fields])) AND “Iran”[All Fields]). Google Scholar: “Irritable bowel syndrome”, “IBS”, and “Iran”	("Irritable bowel syndrome" OR IBS) AND Iran	Exact: PubMed search was described with details of Automatic Term Mapping	10-3-2012		5070	57	18	13	8

First author	Original search description	Search strategy	Changes	Original search date	starting year	Hits GS	Hits PubMed	Included items	Includes in GS (1000)	Includes in PubMed
Javan	(“excisional biopsy” OR “prior Biopsy” OR “previous biopsy” OR “surgical biopsy” OR “open biopsy” OR “excision biopsy” OR “biopsy method” OR “biopsy technique” OR “detection failure” OR nonvisualization OR “non-visualization”) AND “breast cancer” AND sentinel	("excisional biopsy" OR "prior Biopsy" OR "previous biopsy" OR "surgical biopsy" OR "open biopsy" OR "excision biopsy" OR "biopsy method" OR "biopsy technique" OR "detection failure" OR nonvisualization OR non-visualization) AND "breast cancer" AND sentinel	Exact	1-3-2011		6150	137	68	61	29
Navarese	“statins AND/OR diabetes.”	statins AND diabetes	Changed necessary boolean	1-10-2012	1994	84800	3751	17	4	5
Novak	(i) adults; AND (ii) home programme OR home programme OR home exercise program; AND (iii) systematic review OR meta-analysis OR randomized controlled trial OR randomized controlled trial OR clinical trial.	adults AND ("home program" OR "home programme" OR "home exercise program") AND ("systematic review" OR "meta analysis" OR "randomized controlled trial" OR "clinical trial")	Small adaptation, quotes were necessary because of number of hits	1-3-2011		4950	151	30	10	19
Sadeghi	“(penile or penis) and PET,”	(penile OR penis) AND PET	Exact	1-5-2011		2680	44	11	5	5
Susantitaphong	“liver-type fatty acid-binding protein” or “L-FABP.”	"liver-type fatty acid-binding protein" OR L-FABP	Exact	24-9-2012		4130	440	17	16	14

First author	Original search description	Search strategy	Changes	Original search date	starting year	Hits GS	Hits PubMed	Included items	Includes in GS (1000)	Includes in PubMed
Verhoeven	“diabetes” and one of the following words: “telemedicine,” “telecare,” “telehealth,” “e-health,” “teleconsultation,” “telemonitoring,” or “videoconferencing.”	Diabetes AND (telemedicine OR telecare OR telehealth OR e-health OR teleconsultation OR telemonitoring OR videoconferencing)	Rewritten slightly	1-1-2009	1994	31100	410	89	71	67
Wei	"Confusion Assessment Method"	"Confusion Assessment Method"	Exact	1-1-2007		5770	123	25	21	22
Wu	calcium, magnesium and oxaliplatin	Calcium AND magnesium AND oxaliplatin	Added boolean	1-8-2011		5570	40	7	7	7
Chen	(i) ‘pemphigus’; and (ii) ‘human leukocyte anti-gen’, ‘HLA’, ‘major histocompatibility complex’, ‘MHC’ or ‘DRB1’.	Pemphigus AND ("human leukocyte antigen" OR HLA OR "major histocompatibility complex" OR MHC OR DRB1)	Rewritten slightly	30-9-2011		7450	224	18	18	18
Zhu	‘(food OR body OR attention OR emotion) AND (“anorexia nervosa” OR “eating disorder”) AND (“functional magnetic resonance” OR fMRI)’	(food OR body OR attention OR emotion) AND ("anorexia nervosa" OR "eating disorder") AND ("functional magnetic resonance" OR fMRI)	Exact	1-5-2012	1980	3780	94	17	17	14
