# Supplementary material for: The comparative recall of Google Scholar versus PubMed in identical searches for biomedical systematic reviews: a review of searches used in systematic reviews
Source: Syst Rev. 2013 Dec 23;2:115. doi: 10.1186/2046-4053-2-115 (PMC3882110; doi:10.1186/2046-4053-2-115)
Supplement: Additional file 2 — Systematic reviews included in this article. A reference list of all systematic reviews used in this article. [file 2046-4053-2-115-S2.pdf]

Systematic reviews included in this article

1. Belsey JD, Geraint M, Dixon TA: **Systematic review and meta analysis: Polyethylene glycol in adults with non-organic constipation.** *Int J Clin Pract* 2010, **64:**944-955.

2. Bomback AS, Derebail VK, McGregor JG, Kshirsagar AV, Falk RJ, Nachman PH: **Rituximab therapy for membranous nephropathy: a systematic review.** *Clin J Am Soc Nephrol* 2009, **4:**734-744.

3. Cassie S, Menezes C, Birch DW, Shi X, Karmali S: **Effect of preoperative weight loss in bariatric surgical patients: A systematic review.** *Surg Obes Relat Dis* 2011, **7:**760-767.

4. Deeba S, Purkayastha S, Darzi A, Zacharakis E: **Obturator hernias: A review of the laparoscopic approach.** *J Minim Access Surg* 2011, **7:**201-204.

5. Donnenwerth MP, Roukis TS: **Outcome of arthroscopic debridement and microfracture as the primary treatment for osteochondral lesions of the talar dome.** *Arthroscopy J Arthroscopic Relat Surg* 2012, **28:**1902-1907.

6. Espinosa de Los Monteros K, Gallo LC: **The relevance of fatalism in the study of Latinas' cancer screening behavior: a systematic review of the literature.** *Int J Behav Med* 2011, **18:**310-318.

7. Gougoulias N, Khanna A, McBride DJ, Maffulli N: **Management of calcaneal fractures: Systematic review of randomized trials.** *Br Med Bull* 2009, **92:**153-167.

8. Gupta K, Kaur K, Aulakh BS, Kaushal S: **Fesoterodine for overactive bladder: A review of the literature.** *Curr Ther Res Clin Exp* 2010, **71:**273-288.

9. Hardefeldt PJ, Eslick GD, Edirimanne S: **Benign thyroid disease is associated with breast cancer: A meta-analysis.** *Breast Cancer Res Treat* 2012, **133:**1169-1177.

10. Hasani-Ranjbar S, Nayebi N, Larijani B, Abdollahi M: **A systematic review of the efficacy and safety of herbal medicines used in the treatment of obesity.** *World J Gastroenterol* 2009, **15:**3073-3085.

11. Jahangiri P, Jazi MS, Keshteli AH, Sadeghpour S, Amini E, Adibi P: **Irritable Bowel Syndrome in Iran: SEPAHAN Systematic Review No. 1.** *Int J Prev Med* 2012, **3:**S1-9.

12. Javan H, Gholami H, Assadi M, Pakdel AF, Sadeghi R, Keshtgar M: **The accuracy of sentinel node biopsy in breast cancer patients with the history of previous surgical biopsy of the primary lesion: Systematic review and meta-analysis of the literature.** *Eur J Surg Oncol* 2012, **38:**95-109.

13. Navarese EP, Buffon A, Andreotti F, Kozinski M, Welton N, Fabiszak T, Caputo S, Grzesk G, Kubica A, Swiatkiewicz I, et al: **Meta-analysis of impact of different types and doses of statins on new-onset diabetes mellitus.** *Am J Cardiol* 2013, **111:**1123-1130.

14. Novak I: **Effective home programme intervention for adults: a systematic review.** *Clin Rehabil* 2011, **25:**1066-1085.

15. Sadeghi R, Gholami H, Zakavi SR, Kakhki VRD, Horenblas S: **Accuracy of 18F-FDG PET/CT for diagnosing inguinal lymph node involvement in penile squamous cell carcinoma: Systematic review and meta-analysis of the literature.** *Clin Nucl Med* 2012, **37:**436-441.

16. Susantitaphong P, Siribamrungwong M, Doi K, Noiri E, Terrin N, Jaber BL: **Performance of urinary liver-type fatty acid-binding protein in acute kidney injury: a meta-analysis.** *Am J Kidney Dis* 2013, **61:**430-439.

17. Verhoeven F, Tanja-Dijkstra K, Nijland N, Eysenbach G, van Gemert-Pijnen L: **Asynchronous and synchronous teleconsultation for diabetes care: a systematic literature review.** *J Diabetes Sci Technol* 2010, **4:**666-684.

18. Wei LA, Fearing MA, Sternberg EJ, Inouye SK: **The Confusion Assessment Method: a systematic review of current usage.** *J Am Geriatr Soc* 2008, **56:**823-830.

19. Wu Z, Ouyang J, He Z, Zhang S: **Infusion of calcium and magnesium for oxaliplatin-induced sensory neurotoxicity in colorectal cancer: A systematic review and meta-analysis.** *Eur J Cancer* 2012, **48:**1791-1798.

20. Yan L, Wang JM, Zeng K: **Association between HLA-DRB1 polymorphisms and pemphigus vulgaris: a meta-analysis.** *Br J Dermatol* 2012, **167:**768-777.

21. Zhu Y, Hu X, Wang J, Chen J, Guo Q, Li C, Enck P: **Processing of food, body and emotional stimuli in anorexia nervosa: A systematic review and meta-analysis of functional magnetic resonance imaging studies.** *Eur Eating Disord Rev* 2012, **20:**439-450.
