# Supplementary material for: The comparative recall of Google Scholar versus PubMed in identical searches for biomedical systematic reviews: a review of searches used in systematic reviews
Source: Syst Rev. 2013 Dec 23;2:115. doi: 10.1186/2046-4053-2-115 (PMC3882110; doi:10.1186/2046-4053-2-115)
Supplement: Additional file 3 — Improvement of search strategies in Google Scholar. A description of the improved search strategies for Google Scholar and the results obtained with them. [file 2046-4053-2-115-S3.pdf]

improvement of search strategies in Google Scholar

Improvement methods:

Strategy used to create Improved search #1:

Basis information used: description of the search query by the original authors.

Regular search strategy in bibliographic databases: using controlled vocabulary description and synonyms mentioned there. When too much synonyms are found (the total length of the search exceeds 256) the searcher choses those synonyms that generate the most hits in combination with the other elements.

Strategy used to create Improved search #2:

Information used: the titles of the included references.

Synonyms used in the titles of the included references describing the elements of the research question are scored for frequency. Those terms that are used most frequently for each element are used to create a search strategy, until the search query reaches maximally 240 characters (256 minus the necessary character positions for field name allintitle).

Hasani

improved search – 19700 – 25 includes retrieved

(obesity|obese|antiobesity|"weight loss"|"lose weight")("traditional medicine"|"folk medicine"|"Medicinal Plants"|" medicinal herbs"|"herbal medicine"|"plant extracts"|"medical anthropology"|"herbal therapy"|"chinese medicine"|ethnopharmacology)

2340000 hits – 37 includes retrieved

(obesity|obese|"weight loss"|adipose|"weight gain"|adiposity|"body compoisition"|"fat storage"|"weight reduction"|appetite|"weight management")(extract|herbal|extracts|supplement|leaves|extracted|fruits|fruit|supplemented)

Novak

improved

("home (based|program|programme|exercise|rehabilitation)")(exercise|rehabilitation)(trial|review|"meta analysis")

#1 → 17000 hits – 12 includes retrieved

(home|unsupervised)(rehabilitation|exercise|"physical activity")(trial|review|"meta analysis"|randomized)

Without limits: 1600000- 9 included references retrieved

The high number of hits and the relatively small number of retrieved includes, may be caused by noise of the word home. In another attempt this word is combined to phrases.

(("home (based|program|programme|exercise|rehabilitation)")|unsupervised)(rehabilitation|exercise|"physical activity")(trial|review|"meta analysis"|randomized)

#2 → 61400 hits – 13 included references retrieved

Verhoeven

Improvement #1

~diabetes(telemedicine|"tele medicine"|telecare|telehealth|"tele health"|"e health"|ehealth|teleconsultation|telemonitoring|videoconferencing|"video conferencing"|"electronic care"| microcomputer|"handheld computer"|iphone|smartphone|telecommunication)

((telemedicine |telehealth |"Internet based"|telecare|"web based"|"mobile phone"|telemedical|videoconferencing|"text messaging"|"e mail"|telephone|"cell Phone"|pda|"e health")(diabetes|diabetic|insulin))| Telediabetes

872000 hits – 72 retrieved

Belsey

Improved search strategy in Google Scholar

(Constipation|dyschezia|“colonic Inertia”)(polyethylene|Polyhydroxyethyl|Polysorbate|Polyoxyethylene|PEG|PEGs)(randomized| randomised|controlled|placebo|placebos|trial)

13300 hits – 15 included

Search #2

("polyethylene glycol"|forlax|peg|PEG4000)(constipation|constipating|laxative)(randomized|placebo|trial|"double blind"|comparison|controlled|"comparative study"|randomised|"cross over"|"clinical evaluation"|"clinical effects"|trials)

14200 hits – 16 included references retrieved

Navarese

Search #1 – 119000 hits = 13 included references retrieved

(statins|"hmg coa"|atorvastatin|fluvastatin|lovastatin|pitavastatin|pravastatin|rosuvastatin|simvastatin)(diabetes|diabetic|hyperglycemia|hyperinsulinemia|hypoglycemia|hypoinsulinemia|"glucose intolerance")

Search #2

Diabetes is hardly present in the title:

(pravastatin|atorvastatin|simvastatin|Rosuvastatin)(diabetes|hypertensive |"Lipid Lowering"|stroke |"cholesterol lowering "|"myocardial infarction"|heart|hypercholesterolemic|Antihypertensive |ischemic|cardiovascular|Coronary|vascular)

104000 hits – 16 included references retrieved
