# Supplementary material for: The comparative recall of Google Scholar versus PubMed in identical searches for biomedical systematic reviews: a review of searches used in systematic reviews
Source: Syst Rev. 2013 Dec 23;2:115. doi: 10.1186/2046-4053-2-115 (PMC3882110; doi:10.1186/2046-4053-2-115)
Supplement: Additional file 4 — Improvement of search strategies in PubMed. A description of the improved search strategies for PubMed and the results obtained with them. [file 2046-4053-2-115-S4.pdf]

Improvement of search strategies in PubMed

Javan

(“Sentinel Lymph Node Biopsy”[mh] OR ((Biopsy[mh] OR biops*[tiab]) AND sentinel*[tiab])) AND (“Breast Neoplasms”[mh] OR breast*[tiab] OR mammar *[tiab]) AND (Validation Studies [pt] OR “Reproducibility of Results”[mh] OR reproducib*[tiab] OR reliab*[tiab] OR validat*[tiab] OR accura*[tiab] OR detection rate*[tiab] OR detection fail*[tiab] OR false negat*[tiab] OR nonvisuali*[tiab] OR visuali*[tiab])

1985 hits – 61 included references retrieved

Hasani

Research question:

efficacy and safety of effective herbal medicines in the management of obesity in humans and animals

Original search strategy

"obesity" AND ("herbal medicine" OR "plant" OR "plant medicinal" OR "medicine traditional")

("Plants, Medicinal"[mh] OR "Phytotherapy"[mh] OR "Ethnobotany"[mh] OR "Ethnopharmacology"[mh] OR "Pharmacognosy"[mh] OR "Medicine, Traditional"[mh] OR plant extracts[mh] OR Medicinal Plant*[tiab] OR Pharmaceutical Plant*[tiab] OR Healing Plant*[tiab] OR Medicinal Herb*[tiab] OR Phytotherap*[tiab] OR Herb Therap*[tiab] OR Ethnobotan*[tiab] OR Ethnopharmacol*[tiab] OR Pharmacognos*[tiab] OR herbal medicin*[tiab] OR Traditional Medicin*[tiab] OR Folk Remed*[tiab] OR Primitive Medicine*[tiab] OR Folk Medicine*[tiab] OR Indigenous Medicine*[tiab] OR Ethnomedicine*[tiab] OR arabic medicine*[tiab] OR unani medicine*[tiab] OR Ayurvedic Medicine*[tiab] OR Siddha Medicine*[tiab] OR Hindu Medicine*[tiab] OR East Asian Medicine*[tiab] OR Oriental Medicine*[tiab] OR Far East Medicine*[tiab] OR kampo medicine*[tiab] OR kanpo medicine*[tiab] OR Mongolian Medicine*[tiab] OR plant extract*[tiab]) AND ("Obesity"[mh] OR "Anti-Obesity Agents"[mh] OR "Body Weight"[mh] OR "Body Mass Index"[mh] OR "Skinfold Thickness"[mh] OR "Bariatrics"[mh] OR obes*[tiab] OR appetite*[tiab] OR Anorectic*[tiab] OR anorexi*[tiab] OR Antiobes*[tiab] OR Weight-Loss[tiab] OR body weight*[tiab] OR body mass*[tiab] OR bmi[tiab] OR quetelet[tiab] OR overweight*[tiab] OR over weight*[tiab] OR weight gain*[tiab] OR weight loss*[tiab] OR Weight Reduc*[tiab] OR Skinfold Thick*[tiab] OR Bariatric*[tiab])

7883 hits – 67 includes retrieved

Verhoeven

Original search strategy:

Diabetes AND (telemedicine OR telecare OR telehealth OR e-health OR teleconsultation OR telemonitoring OR videoconferencing)

("Diabetes Mellitus"[mh] OR "Diabetes Insipidus"[mh] OR "Diabetic Diet"[mh] OR "Glucose Intolerance"[mh] OR diabet*[tiab]) AND ("Telemedicine"[mh] OR Telecommunications[mh:noexp] OR Telephone[mh] OR "Computers"[mh] OR "internet"[mh] OR "Electronic Mail"[mh] OR Telemedicine*[tiab] OR Telehealth*[tiab] OR eHealth*[tiab] OR e Health*[tiab] OR Mobile Health*[tiab] OR telecommunication*[tiab] OR telephon*[tiab] OR Cellular Phone*[tiab] OR Cellular telephone*[tiab] OR Cell Phone*[tiab] OR Mobile Phone*[tiab] OR Text Messag*[tiab] OR texting[tiab] OR computer*[tiab] OR microcomputer*[tiab] OR minicomputer*[tiab] OR handheld*[tiab] OR hand held*[tiab] OR PDA[tiab] OR Personal Digital Assistant*[tiab] OR palmtop*[tiab] OR internet[tiab] OR World wide web[tiab] OR twitter[tiab] OR Electronic Mail*[tiab] OR e mail*[tiab] OR email*[tiab] OR Remote Consultation*[mh])

7150 hits – 81 included references retrieved

Navarese

Original search:

statins AND diabetes – 4132 hits – 5 included references retrieved

Improved search – 5536 hits – 5 included references retrieved

("Hydroxymethylglutaryl-CoA Reductase Inhibitors"[mh] OR "Hydroxymethylglutaryl-CoA Reductase Inhibitors"[pa] OR statin*[tiab] OR Hydroxymethylglutaryl CoA Reductase Inhibitor*[tiab] OR HMG-CoA Reductase Inhibitor*[tiab] OR Hydroxymethylglutaryl-Coenzyme A Inhibitor*[tiab] OR Hydroxymethylglutaryl-Coenzyme A reductase Inhibitor*[tiab] OR Hydroxymethylglutaryl-CoA Inhibitor*[tiab] OR atorvastatin*[tiab] OR fluvastatin*[tiab] OR lovastatin*[tiab] OR pitavastatin*[tiab] OR pravastatin*[tiab] OR rosuvastatin*[tiab] OR simvastatin*[tiab]) AND ("Glucose Metabolism Disorders"[mh] OR Diabetes Insipidus[mh] OR Diabetic Diet[mh] OR Hyperglycemi*[tiab] OR Hyperinsulin*[tiab] OR Hypoglycem*[tiab] OR diabet*[tiab] OR Glucose Intolerance*[tiab])

Novak [2]

Original search strategy:

adults AND ("home program" OR "home programme" OR "home exercise program") AND ("systematic review" OR "meta analysis" OR "randomized controlled trial" OR "clinical trial")

improved search strategy

(home program*[tiab] OR home based*[tiab] OR home exercise*[tiab] OR home rehab*[tiab] OR unsupervis*[tiab] OR without supervis*[tiab]) AND (rehabilitation[mh] OR rehabilitation[sh] OR rehabilit*[tiab] OR exercise[mh] OR Exercise Therapy[mh] OR exercise*[tiab] OR Motor Activity[mh] OR motor activit*[tiab] OR physical activit*[tiab] OR Exercise Movement Techniques[mh]) AND (review[pt] OR meta analysis[pt] OR review[tiab] OR meta analysis[tiab] OR clinical trial[pt] OR randomized[tiab] OR placebo[tiab] OR randomly[tiab] OR trial[tiab] OR groups[tiab])

4691 hits – 26 included references

Gupta

Original search: Fesoterodine AND overactive bladder AND muscarinic antagonists

The original search seems to contain a duplication of elements. Fesoterodine in the MeSH database is explicitely as “a muscarinic antagonist for treatment of overactive bladder”. The authors could have only searched for the word Fesoterodine, which only returns 113 hits.

Improved PubMed

(fesoterodine[nm] OR fesoterodine*[tiab])

– 113 hits – 12 included references retrieved

Hardefeldt

Original search

("thyroid disease" OR "hyperthyroid" OR "hypothyroid" OR "thyroiditis" OR "graves") AND ("breast disease" OR "breast carcinoma" OR "breast cancer" OR "Breast Diseases ")

222 hits- 18 includes retrieved

("Thyroid Diseases "[mh] OR Thyroid Disease*[tiab] OR goiter[tiab] OR hyperthyroid*[tiab] OR hypothyroid*[tiab] OR Thyroiditis*[tiab] OR Thyrotoxicosis[tiab] OR graves[tiab]) AND (Breast Diseases[mh] OR breast disease*[tiab] OR Breast neoplas*[tiab] OR breast tumo*[tiab] OR breast cancer*[tiab] OR breast carcinom*[tiab] OR Mammary Carcinoma*[tiab] OR Mammary Neoplas*[tiab] OR Cancer of the Breast*[tiab] OR Cancer of Breast*[tiab])

2021 hits – 23 includes retrieved
